# The Inhibitory Effects of Slow-Releasing Hydrogen Sulfide Donors in the Mechanical Allodynia, Grip Strength Deficits, and Depressive-Like Behaviors Associated with Chronic Osteoarthritis Pain

**DOI:** 10.3390/antiox9010031

**Published:** 2019-12-29

**Authors:** Gerard Batallé, Laura Cabarga, Olga Pol

**Affiliations:** 1 Grup de Neurofarmacologia Molecular, Institut d’Investigació Biomèdica Sant Pau, Hospital de la Santa Creu i Sant Pau, 08041 Barcelona, Spain; 2 Institut de Neurociències, Universitat Autònoma de Barcelona, 08193 Barcelona, Spain

**Keywords:** analgesia, anxiety, depression, grip strength, hydrogen sulfide donors, inflammation, microglia, osteoarthritis pain, oxidative stress

## Abstract

Osteoarthritis and its associated comorbidities are important clinical problems that have a negative impact on the quality of life, and its treatment remains unresolved. We investigated whether the systemic administration of slow-releasing hydrogen sulfide (H_2_S) donors, allyl isothiocyanate (A-ITC) and phenyl isothiocyanate (P-ITC), alleviates chronic osteoarthritis pain and the associated emotional disorders. In C57BL/6 female mice with osteoarthritis pain induced by the intra-articular injection of monosodium iodoacetate, we evaluated the effects of repeated administration of A-ITC and P-ITC on the (i) mechanical allodynia and grip strength deficits; (ii) emotional conducts; and (iii) glial activity and expression of inducible nitric oxide synthase (NOS2), phosphatidylinositol 3-kinase (PI3K)/protein kinase B (Akt), and antioxidant enzymes (heme oxygenase 1, NAD(P)H:quinone oxidoreductase-1, glutathione S-transferase mu 1 and alpha 1) in the hippocampus. The administration of A-ITC and P-ITC inhibited the mechanical allodynia, the grip strength deficits, and the depressive-like behaviors accompanying osteoarthritis. Both treatments inhibited microglial activation, normalized the upregulation of NOS2 and PI3K/p-Akt, and maintained high levels of antioxidant/detoxificant enzymes in the hippocampus. Data suggest that treatment with low doses of slow-releasing H_2_S donors might be an interesting strategy for the treatment of nociception, functional disability, and emotional disorders associated with osteoarthritis pain.

## 1. Introduction

Osteoarthritis is one of the most prevalent diseases affecting more than 100 million people worldwide. Osteoarthritis is a chronic degenerative joint disorder characterized by the destruction of articular cartilage causing subchondral bone alterations, inflammation, and intense pain [1]. Chronic osteoarthritis pain, which is characterized by persistent pain with inflammatory and neuropathic components, causes a physical inability to perform daily tasks, difficulty walking, etc. [2]. It is also accompanied by affective disorders such as anxiety and depression, which further contribute to the impairment in the quality of life of patients and exert a negative influence on the perception of pain, creating a vicious circle that leads to unwanted results [3].

At present, the treatment of chronic osteoarthritis pain remains a challenge. Therapies to treat osteoarthritis pain are limited, with modest efficacy and important adverse effects. Furthermore, it is important to note that while some treatments may relieve pain, few are able to reduce the emotional disorders associated with chronic osteoarthritis pain. Therefore, an investigation of new strategies that effectively relieve chronic osteoarthritis pain and the associated comorbidities, such as anxiety and depression, is essential.

Hydrogen sulfide (H_2_S), together with nitric oxide and carbon monoxide, is an integral part of the triad of neurotransmitter gases with important physiological and pathophysiological functions. H_2_S is widely distributed in the central and peripheral nervous system and plays an important role in redox balance, apoptosis, and inflammatory processes [4]. However, despite numerous studies revealing that the exogenous administration of H_2_S exerts powerful anti-inflammatory and antioxidant actions [5], the role of H_2_S in pain modulation is controversial. Thus, conflicting data suggest that H_2_S can be both pronociceptive and antinociceptive, depending on the type of H_2_S-releasing substances used [6]. That is, compounds that release large amounts of H_2_S in a short time and in an uncontrolled manner can relieve pain, not alter it, or even increase it, depending on the dose, the route of administration, and the time of the evaluation of their effects [7,8,9,10]. In contrast, more recent studies show that the administration of substances that are capable of releasing H_2_S slowly, simulating the conditions of H_2_S release in vivo, in addition to exerting potent anti-inflammatory effects [11], are also able to relieve neuropathic pain induced by the administration of antineoplastic drugs in animals [12]. Nevertheless, the effect of the administration of low doses of two slow-releasing H_2_S compounds, such as allyl isothiocyanate (A-ITC) and phenyl isothiocyanate (P-ITC), on the mechanical allodynia and grip strength deficits induced by chronic osteoarthritis in mice has not yet been studied.

Several studies also show that the exogenous administration of H_2_S exerts anxiolytic and antidepressant effects in different animal models of anxiety and depression [13], as well as in the anxiety-like behavior associated with diabetes [14], without affecting locomotor activity. Thus, these data suggest that treatment with slow-releasing H_2_S compounds may be a good approximation for the treatment of the anxiety-like and depressive-like behaviors associated with chronic osteoarthritis pain.

The mechanisms implicated in the development of osteoarthritis are not completely known. Inflammation and oxidative stress are closely integrated in the osteoarthritis pathology. The levels of several inflammatory mediators, such as interleukin-1β (IL-1β) and tumor necrosis factor α (TNFα), are augmented in the joint tissue of osteoarthritis patients [15]. Moreover, IL-1β and TNFα also stimulate the synthesis of prostaglandins, nitric oxide, phosphatidylinositol 3-kinase (PI3K)/protein kinase B (Akt), nuclear factor κB (NF-κB), and diverse mitogen-activated protein kinase (MAPK) signaling pathways further contributing to osteoarthritis pain [16]. Accordingly, the PI3K/Akt/NF-κB and MAPK signaling pathways are activated in the spinal cord and different brain areas, such as the hippocampus and/or prefrontal cortex, of animals with chronic pain [17,18], and their inhibition reduced the development of osteoarthritis and neuropathic pain [19,20].

Microglia are likewise implicated in the progression of chronic pain, and their activation in the supraspinal areas is in part responsible for the molecular neuroplasticity changes associated with chronic pain [21] as well as the emotional disorders accompanying it [3]. Thus, microglial activation has been demonstrated in the medial prefrontal cortex and the hippocampus of animals with depressive-like conducts associated with osteoarthritis or neuropathic pain [18,22] as well as in several brain regions of depressive patients suffering chronic pain [23]. Consequently, the depressive-like behaviors accompanying chronic pain diminished with the administration of microglial inhibitors [24,25].

Oxidative stress also plays an essential role in the pathogenesis of osteoarthritis. High levels of reactive oxygen species have been demonstrated in the synovial tissue, chondrocytes, and fibroblasts of the joint, thus promoting the structural and functional damage of the cartilage [26], which can also potentiate the inflammatory responses by activating the production of nitric oxide synthetized by inducible nitric oxide synthase (NOS2). Therefore, in addition to the well-known positive effects of anti-inflammatory agents, treatment with antioxidant compounds, for example, inducers of nuclear factor erythroid 2-related factor 2 (Nrf2) and/or other enzymes activated by Nrf2, such as heme oxygenase 1 (HO-1), also attenuates the development of osteoarthritis by regulating redox homeostasis [27,28]. Nevertheless, the effects of knee osteoarthritis and those of the systemic treatment with slow-releasing H_2_S compounds in the endogenous antioxidant system in the central nervous system have not been evaluated.

Lastly and taking account that women are more likely to suffer chronic pain than men and because most of the pain symptoms and disorders associated with chronic osteoarthritis pain are more prevalent in women [29], this study was performed in female mice.

In female mice with chronic osteoarthritis pain induced by the intra-articular administration of monosodium iodoacetate (MIA), at 29 days after injection, we assessed the effects of the repeated administration of 4.4 µmol/kg A-ITC or 13.3 µmol/kg P-ITC on (1) the mechanical allodynia; (2) the grip strength deficits; (3) the emotional behaviors associated with chronic osteoarthritis pain; (4) the inflammatory and molecular changes induced by MIA in the central nervous system by evaluating the protein levels of CD11b/c (a microglial marker), glial fibrillary acidic protein (GFAP, an astroglial marker), NOS2, and PI3K/p-Akt; and (6) the expression of antioxidant and detoxificant proteins such as HO-1, NAD(P)H:quinone oxidoreductase-1 (NQO1), glutathione S-transferase mu 1 (GSTM1), and glutathione S-transferase alpha 1 (GSTA1) in the hippocampus.

## 2. Materials and Methods

### 2.1. Animals

We used female mice of 6–8 weeks of age acquired from Envigo laboratories (Barcelona, Spain). All mice weighed between 21 and 25 g and were accommodated in a room with 12/12 h of light/dark and a controlled temperature of 22 °C and humidity of 66%. The animals had free access to food and water, and experiments started at 7 days after acclimatization to the housing conditions. All the proposed experiments were conducted between 9:00 a.m. and 5:00 p.m. and were carried out in accordance with the guidelines of the European Commission’s directive (2010/63/EC) and the Spanish Law (RD 53/2013) regulating animal research, and they were approved by the local Committee of Animal Use and Care of the Autonomous University of Barcelona (number: 1325R5). Maximum efforts were made to minimize the number of animals used and their suffering.

### 2.2. Induction of Osteoarthritis Pain

Osteoarthritis pain was induced in anesthetized mice with isoflurane (2%) by the intra-articular injection of MIA (Sigma-Aldrich, St. Louis, MO, USA). The right knee joint was shaved and flexed at a 90° angle, and 10 µl of MIA (15 mg/mL) dissolved in saline solution (NaCl 0.9%; SS) was intra-articularly injected with a 30-gauge needle. Control mice received the same volume of SS.

### 2.3. Mechanical Allodynia

Mechanical allodynia was evaluated by measuring the hind paw withdrawal response to von Frey filament stimulation. For this purpose, mice were placed in methacrylate cylinders (20 cm high and 9 cm in diameter) on a lifted wire grid through which the von Frey filaments (North Coast Medical, Inc., San Jose, CA, USA) with a bending force range of 0.008 to 3.5 g were applied to each hind paw, using the up–down paradigm reported by Chaplan et al. [30]. The test was started with the 0.4 g filament, and the strength of the next filament was increased or decreased in accordance with the response. Finally, the threshold of the response was calculated from the sequence of filament strength using an Excel program (Microsoft Iberia SRL, Barcelona, Spain) that includes the adjustment of the data curve. Both ipsilateral and contralateral hind paws were assessed, and animals were habituated for 1 h before starting the test to allow appropriate behavioral immobility.

### 2.4. Measurement of Grip Strength

Grip strength was measured with a computerized grip strength meter (Model 47200, Ugo Basile, Varese, Italy) according to the method reported by [31]. To measure grip strength in the hind paws, the experimenter held the animal by the base of the tail, allowing the mice to grasp the metal bar of the grip strength meter with its hind paws. The metal bar was connected to a force transducer that automatically recorded the peak force of each measurement in grams. For each mouse, the grip strength of the hind limbs was measured in triplicate. To prevent the mice from gripping the metal bar with their forepaws during the test, the animals were first allowed to grasp a wire mesh cylinder with their forepaws. Baseline grip strength values were recorded for each mouse as the average of three determinations before the administration of MIA or SS. This value was considered 100% of grip strength and was used as a reference for the following determinations.

### 2.5. Measurement of Anxiety-Like Behaviors

The anxiety-like behaviors were evaluated using the elevated plus maze (EPM) and the open field (OF) tests.

The EPM is an apparatus with 4 arms 5 cm wide and 35 cm long, two of which are open and two closed with walls 15 cm high. The distance of the EPM to the ground is 45 cm. The animal was placed in the central square of the maze facing one of the open arms, and its behavior was recorded by a digital camera for 5 min, according to the method described by [32]. The number of entries in the open and closed arms, as well as the percentage of time spent in the open arms, was calculated for each animal.

The OF test was also used to evaluate the anxiety-like behavior of animals according to the method used by [33]. Mice were placed on a 44 × 44 cm box with a gray nonreflecting base and walls, and their behavior was recorded by a digital camera for 5 min. At the beginning of the test, mice were placed in the center of the arena and were allowed to move freely around the maze and to explore the environment. The number of entries in the central area, the time spent in it, and the number of squares crossed was determined for each animal.

### 2.6. Measurement of Depressive-Like Behaviors

The evaluation of the depressive-like behaviors was realized using the tail suspension test (TST) in accordance with the procedures described by [34] and the forced swimming test (FST) according to the method described by [35].

In the TST, each mouse was suspended 35 cm above the floor with an adhesive tape attached to the tip of its tail. The entire experiment was recorded with a digital camera, and the immobility time was measured over a period of 6 min. The mice were considered immobile when they remained completely quiet.

In the FST, each mouse was placed in a transparent Plexiglas cylinder (25 cm high × 10 cm diameter) containing water to a depth of 10 cm at 24 °C ± 0.1 °C. Each animal was subjected to forced swimming for 6 min, and the total duration of immobility was measured during the last 4 min, when mice show a sufficiently stable level of immobility.

All the behavioral experiments were executed by an experimenter blinded to the treatment applied.

### 2.7. Western Blot Analysis

Animals were euthanized by cervical dislocation at 29 days after injection (MIA or SS). Tissues from the contralateral hippocampus were extracted quickly after killing, frozen, and maintained at −80°C until use. The protein levels of CD11b/c, GFAP, NOS2, PI3K, p-Akt, HO-1, NQO1, GSTM1, and GSTA1 were analyzed. The homogenization of the tissues was performed in ice-cold lysis buffer (50 mM Tris-Base, 150 nM NaCl, 1% NP-40, 2 mM EDTA, 1 mM phenylmethylsulfonyl fluoride, 0.5 Triton X-100, 0.1% sodium dodecyl sulfate, 1 mM Na3VO4, 25 mM NaF, 0.5% protease inhibitor cocktail, 1% phosphatase inhibitor cocktail). All reagents were acquired from Sigma-Aldrich (St. Louis, MO, USA), except for NP-40, which was purchased from Calbiochem (Darmstadt, Germany). After solubilization of crude homogenate for 1 h at 4 °C, it was sonicated for 10 s and centrifuged at 4 °C for 15 min at 700× *g*.

Then, the supernatants (60 µg of total protein) were mixed with 4 × Laemmli loading buffer and loaded onto 4% stacking/10% separating sodium dodecyl sulfate polyacrylamide gels. Proteins were electrophoretically transferred onto a polyvinylidene fluoride membrane for 120 min and blocked with phosphate-buffered saline plus 5% nonfat dry milk or Tris-buffered saline with Tween 20 plus 5% nonfat dry milk or 5% bovine serum albumin for 1 h and 15 min and then incubated with specific rabbit primary anti CD11b/c (1:200), GFAP (1:5000), GSTM1 (1:150), and GSTA1 (1:150) from Novus Biologic, Litton, CO, (USA); NOS2 (1:200) and PI3K (1:200) from Abcam, Cambridge (United Kingdom); p-Akt (1:200) and Akt (1:200) from Cell Signaling Technology, Danvers, MA (USA); HO-1 (1:150) from Enzo Life Sciences, Lausen (Switzerland); and NQO1 (1:250) from Sigma-Aldrich, St. Louis, MO (USA) antibodies overnight at 4 °C. Blots were incubated for 1 h at room temperature with a horseradish peroxidase-conjugated anti-rabbit secondary antibody (GE Healthcare, Little Chalfont, United Kingdom) to detect proteins, which were then visualized by chemiluminescence reagents (ECL kit; GE Healthcare, Little Chalfont, United Kingdom) and exposure to Kodak film. Densitometric analysis was done using Image-J program (National Institutes of Health, Bethesda, MD, USA). We used a rabbit anti glyceraldehyde-3-phosphate dehydrogenase (GAPDH) antibody (1:5000; Merck, Billerica, MA, USA) as a loading control.

### 2.8. Experimental Procedures

In the first experiments, baseline responses for the von Frey filaments and grip strength were established. After that, osteoarthritis pain was induced, and animals were tested again on days 19, 20, 22, 25, 26, 27, and 29 after MIA injection. SS-injected mice were used as controls (six animals per group).

In other groups of animals, we investigated the effects of the intraperitoneal daily administration of 4.4 µmol/kg A-ITC or vehicle from day 25 to day 29 after MIA or SS injection and the effects of the intraperitoneal daily administration of 13.3 µmol/kg P-ITC or vehicle from day 19 to day 29 after MIA or SS injection on the mechanical allodynia and the grip strength deficits induced by MIA (*n* = six animals per group). The effects of A-ITC or the vehicle were evaluated on days 26, 27, and 29 post-MIA or SS injection, while the effects of P-ITC or the vehicle were measured on days 20, 22, 25, and 29 post-MIA or SS injection at 30 min after drug or vehicle injection.

We also evaluated the effects of the treatment with 4.4 µmol/kg A-ITC or vehicle during 4 consecutive days (25 to 29 after MIA or SS injection) and the effects of 13.3 µmol/kg P-ITC or vehicle administered during 10 consecutive days (19 to 29 after MIA or SS injection) on the anxiety- and depressive-like behaviors associated with chronic osteoarthritis pain at 29 days after MIA injection. The anxiety-like behaviors were evaluated in the EPM and OF tests and the depressive-like behaviors in the TST and FST (*n* = 10 animals per group).

The involvement of Kv7 potassium channels in the inhibition of the allodynia, grip strength deficits, and depressive-like behaviors produced by the administration of 4.4 µmol/kg A-ITC or 13.3 µmol/kg P-ITC during 4 days or 10 consecutive days was also studied by evaluating the reversion of these effects with the administration of 8.0 µmol/kg of the selective Kv7 potassium channel blocker, XE-991 [36].

Finally, at day 29 after MIA injection and at 4 (A-ITC) or 10 days (P-ITC) of drug or vehicle administration, mice were euthanized by cervical dislocation, and the protein levels of CD11b/c, GFAP, NOS2, PI3K, p-Akt, HO-1, NQO1, GSTM1, and GSTA1 in the hippocampus were evaluated by Western blot. In these experiments, SS-vehicle-treated mice were used as controls (n = 4 samples per group).

### 2.9. Drugs

A-ITC and P-ITC, obtained from Sigma-Aldrich (St. Louis, MO, USA) and XE-991, purchased in Tocris Bioscience (Ellisville, MO, USA) were dissolved in SS. All drugs were freshly prepared before use and intraperitoneally administered in a final volume of 10 mL/kg, 30 min, and 45 min before testing, in accordance with our preliminary studies and other work [12]. For each group treated with a drug, the respective control group received the same volume of vehicle.

### 2.10. Statistical Analyses

All data are expressed as the mean values ± standard error of the mean (SEM). The statistical results indicate the F value, the degrees of freedom F*x*,*y*, and the *p* value of the ANOVA. Statistical analysis was carried out using the SPSS program (version 13 for Windows, IBM, Madrid, Spain). We used the three-way repeated measures analysis of variance (ANOVA) with injection, treatment, and time as the factors of variation, followed by one-way ANOVA and the Student–Newman–Keuls test to evaluate the effects of the repetitive treatment with A-ITC and P-ITC and their corresponding vehicle on the mechanical allodynia and grip strength deficits induced by MIA.

The effects of the repetitive treatment with A-ITC and P-ITC on the anxiety-like and depressive-like behaviors associated with osteoarthritis pain were assessed using a two-way ANOVA followed by the corresponding one-way ANOVA and the Student–Newman–Keuls test. The reversion of the antinociceptive and antidepressant effects of A-ITC and P-ITC with XE-991were evaluated using a one-way ANOVA and the Student–Newman–Keuls test.

Variations in the protein levels were also analyzed with a one-way ANOVA followed by the Student–Newman–Keuls test. A value of *p* < 0.05 was considered significant.

## 3. Results

### 3.1. Treatment with A-ITC Reverses the Mechanical Allodynia and the Grip Strength Deficits Induced by the Intra-Articular Injection of MIA in Mice

**Figure 1:**
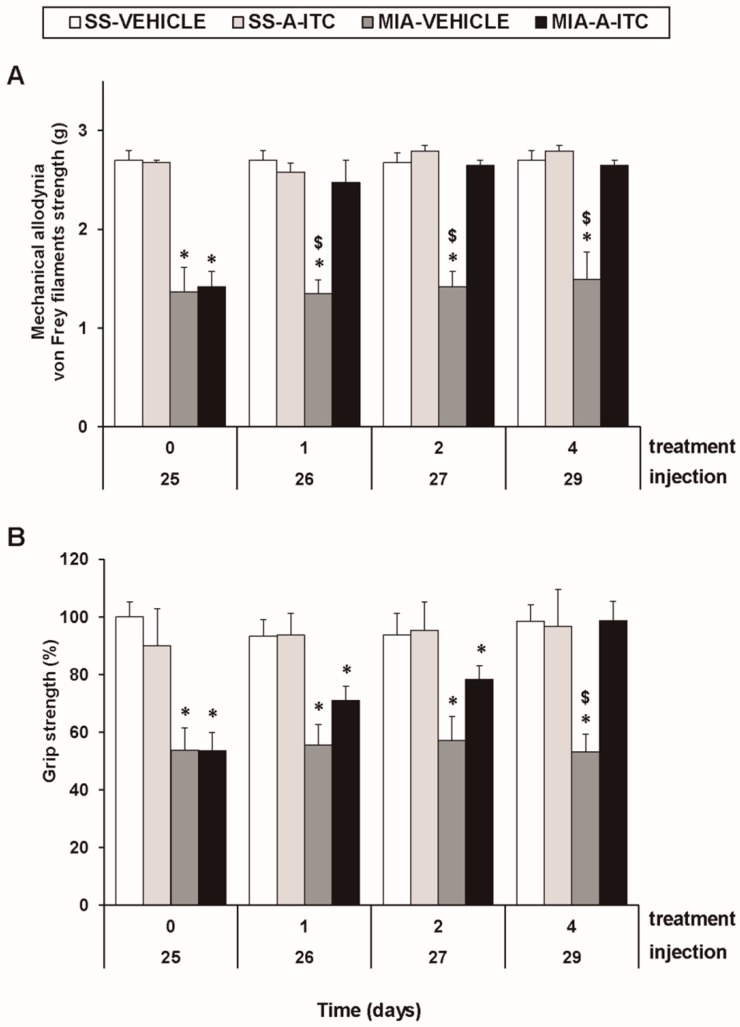
Treatment with allyl isothiocyanate (A-ITC) reduces the mechanical allodynia and the grip strength deficits induced by the intra-articular injection of MIA. The development of (**A**) mechanical allodynia in the ipsilateral paw and (**B**) grip strength deficits in the hind paws of the MIA- or SS-injected mice treated with A-ITC or vehicle for 4 consecutive days are shown. The effects of A-ITC were evaluated at days 26, 27, and 29 after MIA or SS injection. For each test and time evaluated, * denotes significant differences vs. their respective SS-injected mice, and $ denotes significant differences vs. MIA-injected mice treated with A-ITC (*p* < 0.05; one-way ANOVA followed by the Student–Newman–Keuls test). The results are shown as the mean values ± SEM; *n* = 6 animals per experimental group.

The three-way repeated measures ANOVA revealed significant effects among the injection (*F_1,5_* = 211.56, *p* < 0.001), treatment (*F_1,5_* = 50.67, *p* < 0.001), and time (*F_3,15_* = 5.37, *p* < 0.010), and interactions between injection and treatment (*F_1,5_* = 79.12, *p* < 0.001), injection and time (*F _3,15_* = 5.51, *p* < 0.009), treatment and time (*F_3,15_* = 4.26, *p* < 0.023), and among the three factors (*F_3,15_* = 3.41, *p* < 0.045) for the mechanical allodynia. These results confirmed that MIA injection reduces the threshold of the ipsilateral hind paw withdrawal to the von Frey filament stimulation from days 25 to 29 after MIA injection (*p* < 0.001, one-way ANOVA versus the corresponding SS-injected mice treated with vehicle; Figure 1A, Table 1). The administration of A-ITC decreased the mechanical allodynia induced by MIA after one day of treatment. Thus, the threshold of the ipsilateral paw withdrawal to a mechanical stimulus in MIA-injected mice treated with A-ITC was similar to that obtained in SS-injected animals treated with vehicle or A-ITC for one day (Figure 1A). The intraperitoneal administration of A-ITC or vehicle did not produce any significant effect on the mechanical allodynia in either the ipsilateral paw of the SS-injected mice (Figure 1A) or the contralateral paw of the MIA-injected or SS-injected animals (data not shown).

In the grip strength test, the three-way repeated measures ANOVA also revealed significant effects of the injection (*F_1,5_* = 55.69, *p* < 0.001) and time (*F_3,15_* = 3.75, *p* < 0.034) and the interaction between injection and treatment (*F_1,5_* = 21.42, *p* < 0.006). The decreased hind limb grip strength induced by MIA from days 25 to 29 after injection (*p* < 0.009, one-way ANOVA versus the corresponding SS-injected mice treated with vehicle; Figure 1B, Table 1) was inhibited by A-ITC treatment in a time-dependent manner. The grip strength deficits induced by MIA were completely reversed after 4 days of treatment with A-ITC (Figure 1B).

### 3.2. Treatment with P-ITC Reverses the Mechanical Allodynia and the Grip Strength Deficits Induced by the Intra-Articular Injection of MIA in Mice

**Figure 2:**
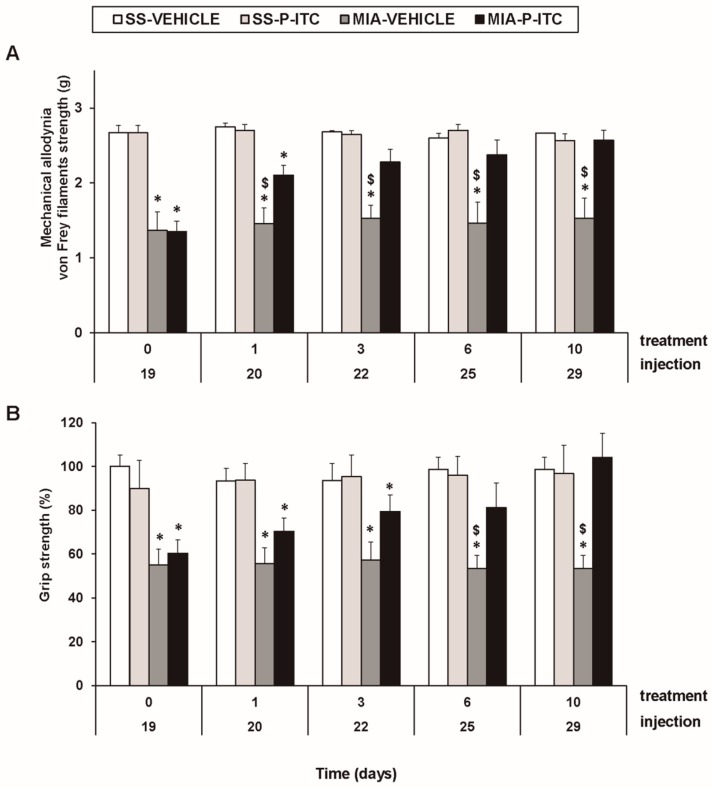
Treatment with phenyl isothiocyanate (P-ITC) decreases the mechanical allodynia and the grip strength deficits induced by the intra-articular injection of MIA. The development of (**A**) the mechanical allodynia in the ipsilateral paw and (**B**) the grip strength deficits in the hind paws of the MIA- or SS-injected mice treated with P-ITC or vehicle for 10 consecutive days are presented. The effects of P-ITC were assessed at days 20, 22, 25, and 29 after MIA or SS injection. For each test and time evaluated, * denotes significant differences vs. their respective SS-injected mice, and $ denotes significant differences vs. MIA-injected mice treated with P-ITC (*p* < 0.05; one-way ANOVA followed by the Student–Newman–Keuls test). The results are shown as the mean values ± SEM; *n* = 6 animals per experimental group.

For mechanical allodynia, the three-way repeated measures ANOVA demonstrated significant effects of the injection (*F_1,5_* = 679.25, *p* < 0.001) and treatment (*F_1,5_* = 12.58, *p* < 0.016). A significant interaction among injection and treatment (*F_1,5_* = 6.59, *p* < 0.050), injection and time (*F_4.20_* = 3.35, *p* < 0.030), and the three factors (*F_4.20_* = 3.54, *p* < 0.024) was also demonstrated. As a consequence, the reduced threshold of the ipsilateral hind paw withdrawal to von Frey filament stimulation from days 19 to 29 after MIA injection (*p* < 0.001, one-way ANOVA versus the corresponding SS-injected mice treated with vehicle; Figure 2A, Table 2) was completely reversed after 3 days of treatment with P-ITC. The intraperitoneal administration of P-ITC did not have any significant effects on the mechanical allodynia in either the ipsilateral paw of the SS-injected mice (Figure 2A) or the contralateral paw of the MIA-injected or SS-injected animals (data not shown).

In the grip strength test, the three-way repeated measures ANOVA also revealed a significant effect of the injection (*F_1,5_* = 53.18, *p* < 0.001) and treatment (*F_1,5_* = 6.06, *p* < 0.050) and the interaction between injection and treatment (*F_1,5_* = 20.10, *p* < 0.006). The grip strength deficits induced by MIA from days 19 to 29 after injection (*p* < 0.015, one-way ANOVA versus the corresponding SS-injected mice treated with vehicle; Figure 2B, Table 2) were inhibited by P-ITC treatment in a time-dependent manner. The decrease in the grip strength induced by MIA was completely reversed after 10 days of treatment with P-ITC (Figure 2B).

### 3.3. The Administration of A-ITC or P-ITC did not Inhibit the Anxiety-Like Behaviors Associated with Chronic Osteoarthritis Pain in Mice

The effects of A-ITC and P-ITC treatments on the anxiety-like behaviors accompanying osteoarthritis pain were evaluated in the EPM and OF tests at 29 days after the intra-articular injection of MIA.

**Figure 3:**
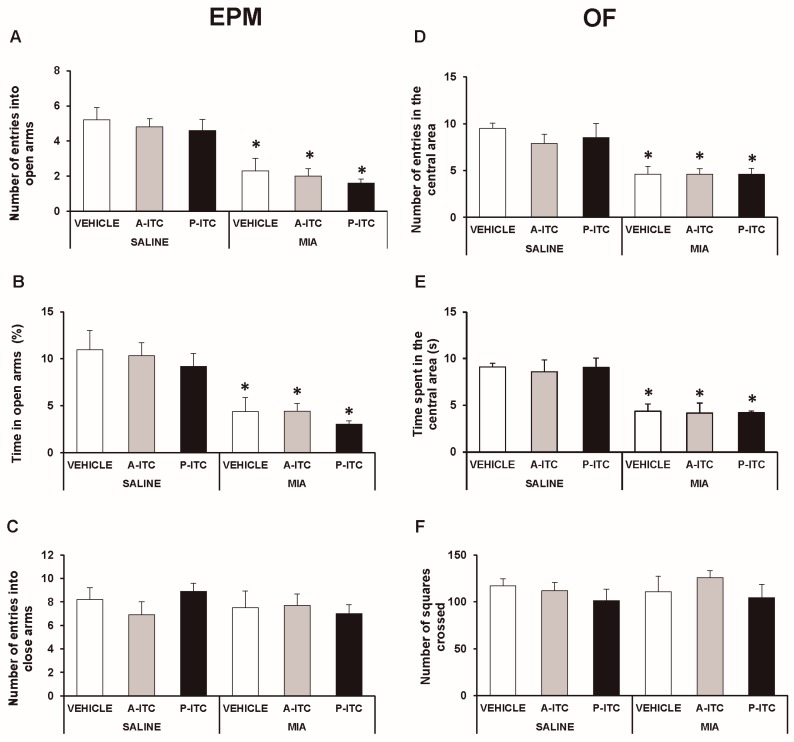
**Treatment with A-ITC or P-ITC does not alter the anxiety-like behaviors associated with chronic osteoarthritis pain.** The anxiety-like behaviors were evaluated on day 29 after MIA or SS injection and at 4 or 10 days of treatment with A-ITC or P-ITC in the elevated plus maze (EPM) and open field (OF) tests. In the EPM, (**A**) the number of entries into the open arms, (**B**) percentage of time spent in the open arms, and (**C**) the number of entries into the closed arms are shown. For OF, (**D**) the number of entries into the central area, (**E**) time spent in the central area (s), and (**F**) the number of squares crossed are shown. For each test evaluated, * denotes significant differences vs. their respective SS-injected mice (*p* < 0.05; one-way ANOVA followed by the Student-Newman-Keuls test). The results are presented as the mean ± SEM; *n* = 10 animals per experimental group.

In the EPM test, the two-way ANOVA revealed significant effects of the injection in the number of entries in the open arms (*F_1,54_* = 49.78, *p* < 0.001) and in the time spent in the open arms (*F_1,54_* = 38.19, *p* < 0.001). The significant reduction in the number of entries (*F_5,54_* = 10.33, *p* < 0.001, one-way ANOVA versus SS-injected mice treated with vehicle; Figure 3A) and in the time spent in the open arms (*F_5,54_* = 7.97, *p* < 0.001, one-way ANOVA versus SS-injected mice treated with vehicle; Figure 3B) observed in MIA-injected mice treated with vehicle demonstrated the anxiety-like behavior associated with chronic osteoarthritis pain. Treatment with A-ITC or P-ITC did not normalize the anxiety-like responses induced by MIA. Moreover, no differences in the number of entries into the closed arms were observed between MIA and SS-injected mice treated with A-ITC, P-ITC, or vehicle (Figure 3C).

In the OF test, the two-way ANOVA also revealed significant effects of the injection in the number of entries in the central area (*F_1,54_* = 35.69, *p* < 0.001) and in the time spent in it (*F_1,54_* = 36.30, *p* < 0.001). The significant reductions in the number of entries into the central area (*F_5,54_* = 7.69, *p* < 0.001, ANOVA versus SS-injected mice treated with vehicle; Figure 3D) and in the time spent in it (*F_5,54_* = 7.30, *p* < 0.001, ANOVA versus SS-injected mice treated with vehicle; Figure 3E) caused by MIA were not reversed by the repeated administration of A-ITC or P-ITC. Thus, confirming that both treatments are not capable of normalizing the anxiety-like responses induced by MIA. Finally, no differences in the number of squares crossed in the OF test were detected between MIA and SS-injected mice treated with A-ITC, P-ITC, or vehicle (Figure 3F).

### 3.4. The Administration of A-ITC and P-ITC Inhibits the Depressive-Like Behaviors Associated with Chronic Osteoarthritis Pain

**Figure 4:**
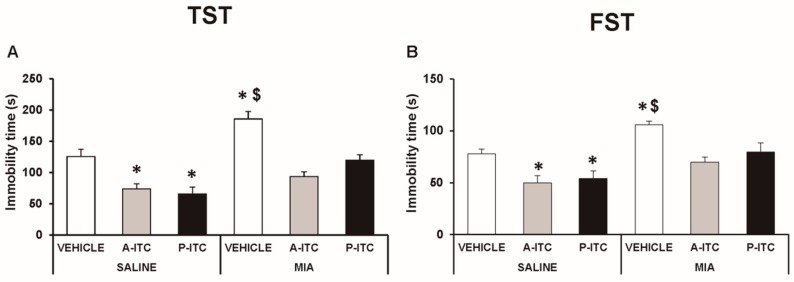
Treatment with A-ITC or P-ITC decreases the depressive-like behaviors associated with chronic osteoarthritis pain. The immobility time (s) evaluated with the (**A**) tail suspension test (TST) and (**B**) forced swimming test (FST) at 29 days after MIA or SS injection in mice treated for 4 consecutive days with A-ITC or for 10 days with P-ITC is shown. For each test evaluated, * denotes significant differences vs. SS-injected mice treated with vehicle, and $ denotes significant differences vs. MIA-injected mice treated with a drug (*p* < 0.05; one-way ANOVA followed by the Student–Newman–Keuls test). The results are presented as the mean values ± SEM; *n* = 10 animals per experimental group.

Depressive-like behaviors were assessed in the TST and FST at 29 days after the intra-articular injection of MIA. The two-way ANOVA revealed significant effects of the injection (*F_1,54_* = 30.16, *p* < 0.001) and treatment (*F_2,54_* = 30.86, *p* < 0.001) in the TST. Thus, osteoarthritis induced a depressive-like behavior evidenced by the significant increase in the immobility time (*F_5,54_* = 19.32, *p* < 0.001, one-way ANOVA versus SS-injected mice treated with vehicle, Figure 4A), which was reduced with the administration of A-ITC or P-ITC. Moreover, a significant effect of the injection (*F_1,54_* = 23.47, *p* < 0.001) and treatment (*F_2,54_* = 10.70, *p* < 0.001) was also observed in the FST, and both treatments reversed the increased immobility time observed in MIA-injected animals (*F_5,54_* = 9.18, *p* < 0.001, one-way ANOVA versus SS-injected mice treated with vehicle, Figure 4B). In the TST and FST, both treatments also reduced the immobility time in SS-injected mice (Figure 4). These results demonstrated the antidepressant effects of these drugs in animals with and without chronic pain.

### 3.5. The Administration of XE-991 Reverses the Inhibition of the Mechanical Allodynia, Grip Strength Deficits, and Depressive-Like Behaviors of A-ITC and P-ITC during Osteoarthritis Pain

**Figure 5:**
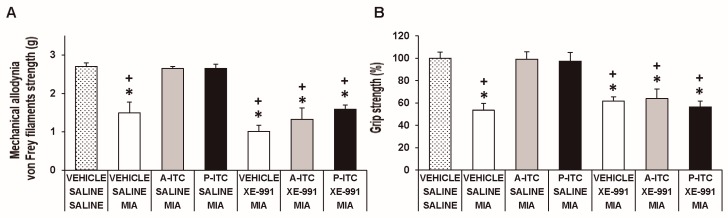
The administration of XE-991 reverses the inhibition of the mechanical allodynia and grip strength deficits of A-ITC and P-ITC during chronic osteoarthritis pain. The mechanical allodynia in the ipsilateral paw (**A**) and grip strength deficits in the hind paws (**B**) of the MIA-injected mice treated with A-ITC or P-ITC during 4 or 10 days alone and combined with the selective Kv7 potassium channel blocker XE-991 are shown. The effects of XE-991 administered alone are also represented. For each test evaluated, * denotes significant differences vs. saline-saline-injected mice treated with vehicle and + denotes significant differences vs. MIA saline-injected mice treated with a drug (*p* < 0.05; one-way ANOVA followed by the Student–Newman–Keuls test). The results are shown as the mean values ± SEM; *n* = 6 animals per experimental group.

**Figure 6:**
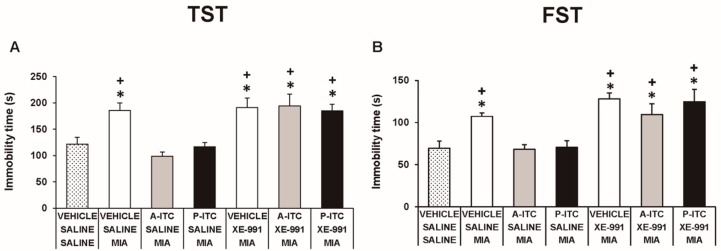
The administration of XE-991 reverses the antidepressant effects of A-ITC and P-ITC in mice with chronic osteoarthritis pain. The immobility time (s) evaluated with the (**A**) TST and (**B**) FST in mice treated for 4 or 10 consecutive days with A-ITC or P-ITC alone and combined with the selective Kv7 potassium channel blocker XE-991 is shown. The effects of XE-991 administered alone are also represented. For each test evaluated, * denotes significant differences vs. saline–saline-injected mice treated with vehicle and + denotes significant differences vs. MIA–saline-injected mice treated with a drug (*p* < 0.05; one-way ANOVA followed by the Student–Newman–Keuls test). The results are shown as the mean values ± SEM; *n* = 6–8 animals per experimental group.

To assess the involvement of H_2_S in the effects produced by the repetitive administration of 4.4 µmol/kg A-ITC during 4 days or 13.3 µmol/kg P-ITC during 10 days in animals with osteoarthitis pain, the reversion of their effects with the selective Kv7 potassium channel blocker, XE-991, administered at 8.0 µmol/kg over one day was evaluated. Our results showed that the administration of XE-991 reversed the inhibition of the mechanical allodynia (F_6,35_ = 16.70, *p* < 0.001, one-way ANOVA versus MIA saline-injected mice treated with vehicle, Figure 5A) and the grip strength deficits induced by A-ITC and P-ITC treatments (F_6,35_ = 11.32, *p* < 0.001, one-way ANOVA versus MIA-saline-injected mice treated with vehicle, Figure 5B). Moreover, the antidepressant effects of A-ITC and P-ITC in the TST (F_6,42_ = 8.63, *p* < 0.001, one-way ANOVA versus MIA saline-injected mice treated with vehicle, Figure 6A) and FST (F_6,42_ = 9.50, *p* < 0.001, one-way ANOVA versus MIA saline-injected mice treated with vehicle, Figure 6B) were reversed with XE-991. The administration of XE-991 alone (Figure 5 and Figure 6) did not produce any significant effect.

### 3.6. Effect of A-ITC and P-ITC on the Expression of CD11b/c, GFAP, NOS2, PI3K, p-Akt, HO-1, NQO1, GSTM1, and GSTA1 in the Hippocampus of MIA-Injected Mice

**Figure 7:**
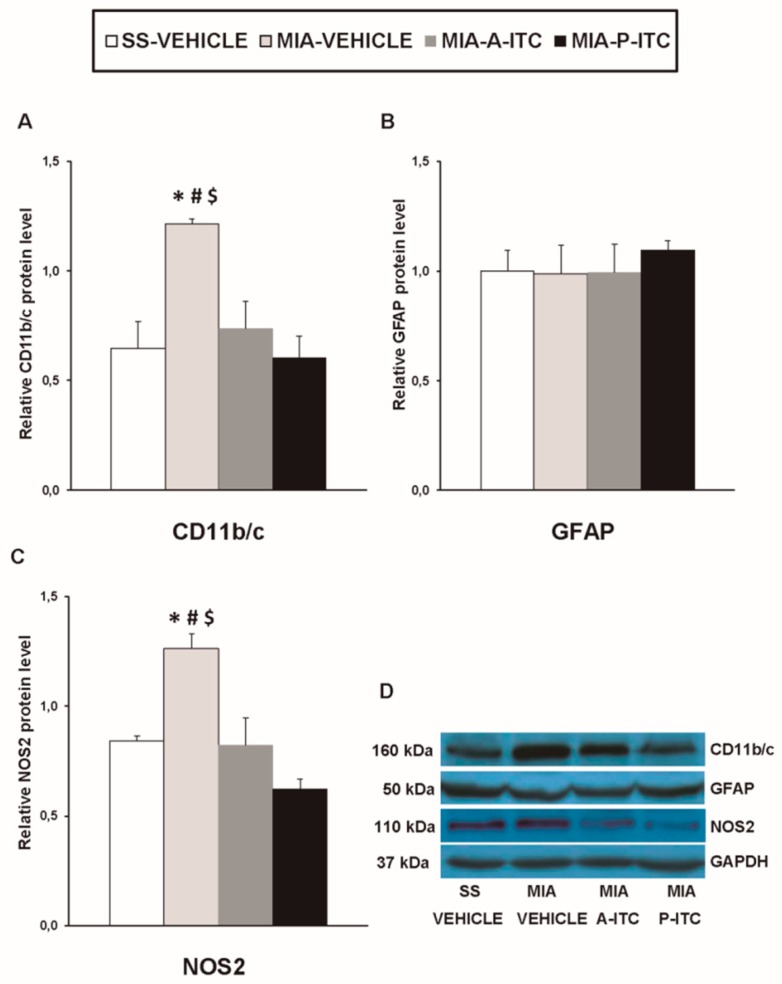
A-ITC and P-ITC treatments inhibit the overexpression of CD11b/c and NOS2 in the hippocampus of mice with osteoarthritis pain. The relative protein levels of (**A**) CD11b/c, (**B**) GFAP, and (**C**) NOS2 in the hippocampus of MIA-injected mice treated with A-ITC, P-ITC, or vehicle are presented. The SS-injected mice treated with vehicle were used as controls. (**D**) Representative blots for CD11b/c (160 kDa), GFAP (50 kDa), NOS2 (110 kDa), and GAPDH (37 kDa). All proteins are expressed relative to GAPDH levels. In all panels, * denotes significant differences vs. SS-injected mice treated with vehicle, # vs. MIA-injected mice treated with A-ITC and $ vs. MIA-injected mice treated with P-ITC (*p* < 0.05; one-way ANOVA followed by the Student–Newman–Keuls test). The results are presented as the mean ± SEM; *n* = 4 samples per experimental group.

**Figure 8:**
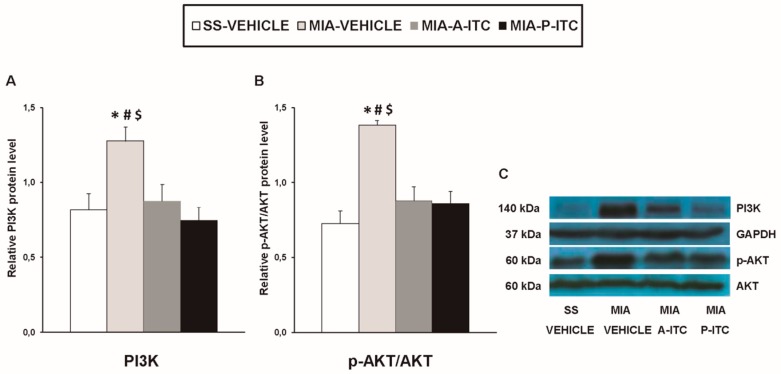
A-ITC and P-ITC treatments inhibit the overexpression of phosphatidylinositol 3-kinase (PI3K) and protein kinase B (p-Akt) in the hippocampus of mice with osteoarthritis pain. The relative protein levels of (**A**) PI3K and (**B**) p-Akt/Akt in the hippocampus of MIA-injected mice treated with A-ITC, P-ITC, or vehicle are presented. The SS-injected mice treated with vehicle were used as controls. (**C**) Representative blots for PI3K (140 kDa), glyceraldehyde-3-phosphate dehydrogenase (GAPDH) (37 kDa), p-Akt (60 kDa), and Akt (60 kDa). PI3K is expressed relative to GAPDH levels and p-Akt relative to total Akt. In all panels, * denotes significant differences vs. SS-injected mice treated with vehicle, # vs. MIA-injected mice treated with A-ITC and $ vs. MIA-injected mice treated with P-ITC (*p* < 0.05; one-way ANOVA and Student–Newman–Keuls test). Results are presented as the mean ± SEM; *n* = 4 samples per experimental group.

**Figure 9:**
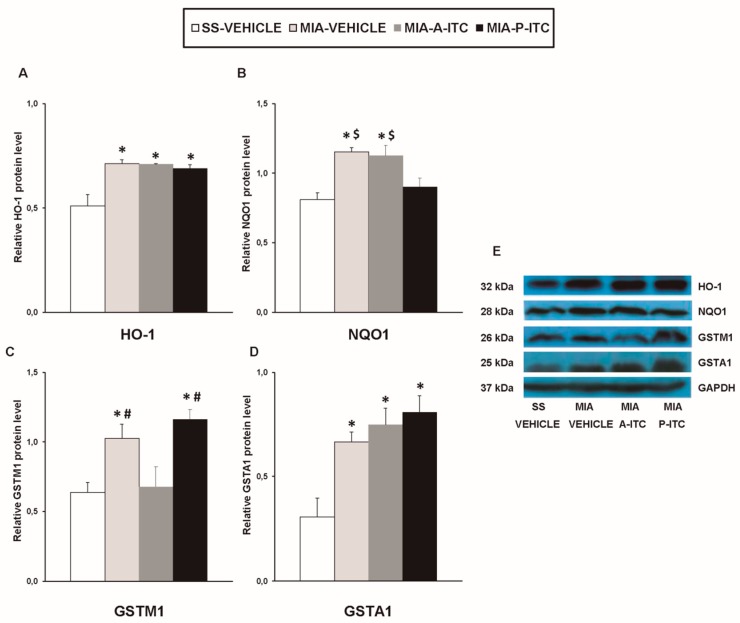
A-ITC and/or P-ITC treatments maintain the overexpression of heme oxygenase 1 (HO-1), quinone oxidoreductase-1 (NQO1), glutathione S-transferase mu 1 (GSTM1), and/or glutathione S-transferase alpha 1 (GSTA1) in the hippocampus of mice with osteoarthritis pain. The relative protein levels of (**A**) HO-1, (**B**) NQO1, (**C**) GSTM1, and (**D**) GSTA1 in the hippocampus of MIA-injected mice treated with A-ITC, P-ITC, or vehicle are presented. The SS-injected mice treated with vehicle were used as controls. (**E**) Representative blots for HO-1 (32 kDa), NQO1 (28 kDa), GSTM1 (26 kDa), GSTA1 (25 kDa), and GAPDH (37 kDa). All proteins are expressed relative to GAPDH levels. In all panels, * denotes significant differences vs. SS-injected mice treated with vehicle, # vs. MIA-injected mice treated with A-ITC and $ vs. MIA-injected mice treated with P-ITC (*p* < 0.05; one-way ANOVA and Student–Newman–Keuls test). The results are presented as the mean ± SEM; *n* = 4 samples per experimental group.

Our results showed that the intra-articular injection of MIA caused a significant increase in the expression of CD11b/c (*F_3,12_* = 4.39, *p* < 0.026, one-way ANOVA; Figure 7A), NOS2 (*F_3,12_* = 13.34, *p* < 0.001, one-way ANOVA; Figure 7C), PI3K (*F_3,12_* = 5.63, *p* < 0.012, one-way ANOVA; Figure 8A), p-Akt (*F_3,12_* = 8.60, *p* < 0.003, one-way ANOVA; Figure 8B), HO-1 (*F_3,12_* = 9.87, *p* < 0.001, one-way ANOVA; Figure 9A), NQO1 (*F_3,12_* = 8.87, *p* < 0.002, one-way ANOVA; Figure 9B), GSTM1 (*F_3,12_* = 6.54, *p* < 0.007, one-way ANOVA; Figure 9C), and GSTA1 (*F_3,12_* = 3.75, *p* < 0.041; Figure 9D) in the hippocampus. Treatment with A-ITC and P-ITC normalized the enhanced protein levels of CD11b/c, NOS2, PI3K, and p-Akt in the hippocampus (Figure 7 and Figure 8). Moreover, while both treatments maintained the increased protein levels of HO-1 and GSTA1 in the hippocampus of MIA-injected mice, the increased expression of NQO1 and GSTM1 induced by MIA was only maintained in A-ITC and P-ITC-treated animals, respectively (Figure 9). No changes in the expression of GFAP were detected in any of the groups evaluated (Figure 7B).

## 4. Discussion

This study reveals that the repeated administration of low doses of A-ITC or P-ITC inhibited the mechanical allodynia and grip strength deficits induced by the intra-articular injection of MIA as well as the depressive-like behaviors associated with chronic osteoarthritis pain. Both treatments also normalized microglial activation, NOS2 overexpression, and PI3K/Akt phosphorylation induced by MIA and maintained high levels of the antioxidant enzymes in the hippocampus of mice with osteoarthritis.

The role of H_2_S in pain modulation is controversial. It can produce pronociceptive or antinociceptive effects depending on the type of H_2_S donor, the doses and pain models used, etc. [6]. Thus, while the fast donors of H_2_S cannot alter or even increase pain [7,8,9,10], more recent studies show that the administration of substances capable of releasing H_2_S slowly, simulating the conditions of H_2_S release in vivo, in addition to exert potent anti-inflammatory effects [11], are also able to relieve neuropathic pain induced by the administration of antineoplastic drugs in animals [12]. In accordance, our results showed that the administration of A-ITC or P-ITC, two slow-releasing H_2_S donors, inhibited the mechanical allodynia induced by the intra-articular administration of MIA with different effectiveness levels. That is, while one day of treatment with 4.4 µmol/kg A-ITC is enough to block the mechanical allodynia induced by osteoarthritis, three days of treatment with 13.3 µmol/kg of P-ITC are required to abolish it. Our results are in agreement with the inhibitory effects of A-ITC and P-ITC in the mechanical allodynia provoked by chemotherapy [12] and showed, for the first time, the antiallodynic properties of these compounds in female mice with chronic osteoarthritis pain at 29 days after MIA injection.

Chronic osteoarthritis pain, in addition to inducing important hypersensitivity in patients, also causes alterations in physical functioning that negatively influence several aspects of daily life, such as difficulty walking, inability to perform daily tasks, etc. [2]. Thus, it is important to measure the effect of treatments on the physical function of the subject, and grip strength evaluation is habitually utilized as a functional measure in patients with joint inflammation. Consequently, in this study, we evaluated the effects of A-ITC and P-ITC treatments on the functional deficits induced by osteoarthritis by measuring the grip strength of these animals. Our results demonstrated that the intra-articular injection of MIA decreased the hind limb grip strength from days 19 to 29 after the induction of osteoarthritis, corroborating and expanding the results obtained at 21 days after MIA injection [37]. Our data also revealed that while in complete Freund’s adjuvant (CFA)-induced joint inflammation the grip strength deficit returns to normal values at 21 days after induction [31], in MIA-induced osteoarthritis, the grip strength deficit remains constant for at least 29 days. These differences may be related to the fact that MIA provokes cartilage destruction, and this effect is more persistent over time. Our results further demonstrated that the repeated administration of A-ITC and P-ITC reduced the grip strength deficits induced by MIA. However, as occurs in mechanical allodynia, their effects are produced at different times according to the treatment. Thus, while 4 days of treatment with 4.4 µmol/kg A-ITC completely reversed the grip strength deficit induced by MIA, 10 days of treatment with 13.3 µmol/kg P-ITC were required to block it. These results suggested that treatment with A-ITC is more effective than treatment with P-ITC at reversing the mechanical allodynia and the grip strength deficits generated by osteoarthritis. Our findings also showed that both compounds produce a more rapid recovery of the mechanical allodynia than the recovery of the grip strength deficits caused by osteoarthritis. These results are in contrast with the equipotency of oxycodone in reversing the allodynia and physical function but agree with the effects produced by other drugs, such as morphine, which is also less potent in the reversion of the grip strength deficits than the mechanical allodynia caused by CFA-induced joint inflammation in female mice [31].

Chronic osteoarthritis pain is also accompanied by emotional disorders such as depression and anxiety. Thus, the evaluation of the effects induced by a treatment on the affective manifestations associated with chronic osteoarthritis pain is fundamental. Several studies have demonstrated that the administration of some H_2_S donors produced anxiolytic effects [13,38]. In this study, we evaluated the possible anxiolytic effects induced by treatment with slow-releasing H_2_S donors in animals with chronic osteoarthritis pain using the EPM and OF tests. Our results confirmed the anxiety-like behaviors induced by the intra-articular injection of MIA, as demonstrated by the reduced number of entries and the time spent in the open arms in the EPM test as well as by the diminished number of entrances into the central area and the time spent in it in the OF test. We further demonstrated that the administration of A-ITC or P-ITC did not alter the anxiety-like behaviors in mice with osteoarthritis pain. These results are in contrast with the anxiolytic effects induced by other fast-releasing H_2_S donors, such as sodium hydrosulfide [13] or sodium sulfide in control animals and diabetic rats [14,38], thus displaying the different properties of H_2_S in accordance with the type of donor used. Finally, treatment with A-ITC or P-ITC did not alter the number of entries into the closed arms in the EPM test or the number of squares crossed in the OF test, revealing that these compounds do not affect the locomotor activity of animals with or without osteoarthritis pain.

Chronic osteoarthritis pain is also associated with the development of depression, which is difficult to treat with conventional analgesics. In agreement with other studies, our results confirmed that osteoarthritis induced by MIA causes depressive-like behaviors [22]. In contrast to anxiety, the administration of A-ITC or P-ITC inhibited the depressive-like responses promoted by osteoarthritis. Thus, the increase in the immobility time observed in the TST and the FST at 29 days after MIA injection was inhibited by treatment with A-ITC or P-ITC. These results demonstrated the antidepressant effects of both slow-releasing H_2_S donors in animals with chronic osteoarthritis pain. Moreover, and in accordance with the antidepressant properties of other H_2_S donors [39], the reduction in the immobility time induced by A-ITC and P-ITC in control mice also evidenced their antidepressant properties in the absence of pain.

In this study, we also evaluated the possible involvement of Kv7 potassium channels in the inhibitory effects of A-ITC and P-ITC by assessing the reversion of their effects with the selective Kv7 potassium channel blocker, XE-991 [36]. Our results showed that the inhibition of the mechanical allodynia, grip strength deficits, and the depressive like-behaviors produced by A-ITC and P-ITC treatments was reversed with the administration of XE-991. These data agree with the reversion of the antinociceptive effects of A-ITC, P-ITC, and other H_2_S donors (NaHS, glucoraphanin, and GYY4137) by selective Kv7 potassium channel blockers during chronic pain [12,40].

It is well recognized that the activation of several brain areas, for example the hippocampus, modulates pain and the emotional manifestations of pain. In this study, we evaluated the effects of the administration of A-ITC and P-ITC on microglial activation and the inflammatory and/or biochemical changes induced by chronic osteoarthritis in this brain region. Microglia play a critical role in the development and maintenance of chronic pain as well as in depression. Several works demonstrated microglial activation in the spinal cord [41] and the medial prefrontal cortex of animals with osteoarthritis at 25–26 days after MIA injection [22]. In this study, the increased expression of CD11b/c promoted by MIA in the hippocampus at 29 days after injection supported these findings and further demonstrated microglial activation in this brain area. Moreover, considering that treatment with minocycline relieves the depressive-like behaviors associated with chronic cancer pain by inhibiting microglia activation in the hippocampus [25]; the inhibition of microglial activation induced by A-ITC and P-ITC might explain their antidepressant effects during chronic osteoarthritis pain. In contrast to microglia, nonchanges in the expression of the astroglial marker (GFAP) were observed in the hippocampus. These results agree with the absence of changes in the spinal astrocyte expression reported by other studies performed in animals with chronic osteoarthritis pain [42,43]. Nonetheless, our treatments did not alter the astrocyte expression in the hippocampus.

Nitric oxide also contributes to the pathophysiology of osteoarthritis. High concentrations of nitric oxide synthesized by NOS2 were detected in the synovial fluid, and treatment with specific NOS2 inhibitors attenuated the osteoarthritis pain induced by MIA by reducing nitric oxide production [44]. Other studies also indicated that the cytoprotective effects of H_2_S in osteoarthritis were mediated via inhibiting nitric oxide synthesis. To elucidate whether NOS2 is also involved in the effects produced by A-ITC and P-ITC during osteoarthritis, we investigated its expression in the hippocampus. Our results showed that the increased protein levels of NOS2 in this supraspinal region of animals with knee osteoarthritis were inhibited by the administration of slow-releasing H_2_S donors. The fact that chronic inflammatory pain is inhibited in mice lacking NOS2 [45] supports the hypothesis that the antinociceptive effects induced by A-ITC and P-ITC during osteoarthritis might also be due to modulating the expression of NOS2 in the hippocampus. Nevertheless and taking into account that several authors have demonstrated that the exposition to H_2_S actives the synthesis of the endothelial nitric oxide synthase (NOS3) in different cell types [46], we cannot discard that the antinociceptive and/or antidepressant effects of the slow-releasing H_2_S donors can be also produced via modulating NOS3.

The intracellular signaling pathway PI3K/p-Akt also regulates the inflammatory processes in the pathogenesis of osteoarthritis [47]. In accordance with the activated PI3K/Akt pathway observed in the spinal cord and hippocampus of animals with chronic neuropathic pain [18], our results further demonstrated that knee osteoarthritis also activates this signaling pathway in the hippocampus, which was inhibited by both A-ITC and P-ITC treatments. Thus, taking account that the inhibition of PI3K/p-Akt alleviates the mechanical allodynia induced by chronic pain [19], the antiallodynic effects of A-ITC and P-ITC might also be mediated via the hippocampal regulation of this pathway.

Finally, it is well known that oxidative stress is another key factor in the onset and progression of osteoarthritis, and the Nrf2 transcription factor activates the expression of several antioxidant and detoxificant enzymes to protect the organism against oxidative stress [48]. Consequently, while the inhibition of Nrf2 or HO-1 results in increased osteoarthritis severity [49], its induction evokes favorable effects during osteoarthritis [50]. In this study, we evaluated the effects of treatment with A-ITC and P-ITC on the protein levels of the antioxidant enzymes HO-1, NQO1, GSTM1, and GSTA1 in the hippocampus of animals with osteoarthritis pain. Our data indicate that the expression of these antioxidant/detoxificant enzymes is significantly increased in the hippocampus of MIA-injected mice and that treatment with A-ITC and P-ITC maintains its high levels in this brain area. These findings are in accordance with the overexpression of Nrf2 and HO-1 detected in the cartilage of patients and animals with osteoarthritis [51,52], suggesting that the high expression of the antioxidant/detoxificant proteins detected in the hippocampus of mice with knee osteoarthritis might act as an endogenous protective response against the oxidative stress generated by this pathology. Furthermore, the fact that treatment with A-ITC and/or P-ITC maintained the elevated levels of HO-1, NQO1, GSTM1, and/or GSTA1 supported the idea that the antinociceptive/anti-inflammatory effects induced by these slow-releasing H_2_S donors during chronic osteoarthritis might also be produced by keeping the endogenous antioxidant system activated.

## 5. Conclusions

In conclusion, this study demonstrated that treatment with A-ITC and P-ITC inhibits the mechanical allodynia and grip strength deficits caused by osteoarthritis as well as the depressive-like behaviors associated with chronic osteoarthritis pain. Both treatments also modulate microglial activation and the inflammatory/nociceptive reactions induced by chronic osteoarthritis in the hippocampus and maintain the endogenous antioxidant system activated. Our data suggest that treatment with low doses of slow-releasing H_2_S donors might be an interesting strategy for the treatment of nociception, functional disability, and depressive-like conducts related to chronic osteoarthritis pain.

## Figures and Tables

**Table 1:** Summary of the one-way ANOVA’s performed with the results obtained for the mechanical allodynia and grip strength deficits at 0, 1, 2, and 4 days after the administration of allyl isothiocyanate (A-ITC) or vehicle in saline (SS) and monosodium iodoacetate (MIA)-injected mice.

Time of Treatment (days)				
	0	1	2	4
Mechanical	*F_3,20_* = 25.15	*F_3,20_* = 18.27	*F_3,20_* = 52.24	*F_3,20_* = 17.98
allodynia	*p* < 0.001	*p* < 0.001	*p* < 0.001	*p* < 0.001
Grip	*F_3,20_* = 7.97	*F_3,20_* = 8.26	*F_3,20_* = 5.05	*F_3,20_* = 7.09
strength	*p* < 0.001	*p* < 0.001	*p* < 0.009	*p* < 0.002

**Table 2:** Summary of the one-way ANOVA’s performed with the results obtained for the mechanical allodynia and grip strength deficits at 0, 1, 3, 6, and 10 days after the administration of P-ITC or vehicle in SS and MIA-injected mice.

Time of Treatment (days)					
	0	1	3	6	10
Mechanical	*F_3,20_* = 21.29	*F_3,20_* = 20.36	*F_3,20_* = 18.33	*F_3,20_* = 9.92	*F_3,20_* = 10.53
allodynia	*p* < 0.001	*p* < 0.001	*p* < 0.001	*p* < 0.001	*p* < 0.001
Grip	*F_3,20_* = 6.84	*F_3,20_* = 7.65	*F_3,20_* = 4.44	*F_3,20_* = 6.27	*F_3,20_* = 6.13
strength	*p* < 0.001	*p* < 0.001	*p* < 0.015	*p* < 0.004	*p* < 0.004
